# Positive end-expiratory airway pressure does not aggravate ventilator-induced diaphragmatic dysfunction in rabbits

**DOI:** 10.1186/s13054-014-0494-0

**Published:** 2014-09-12

**Authors:** Catherine SH Sassoon, Ercheng Zhu, Liwei Fang, Gary C Sieck, Scott K Powers

**Affiliations:** Department of Medicine, Section of Pulmonary and Critical Care Medicine, VA Long Beach Healthcare System, 5901 East 7th Street, Long Beach, CA 90822 USA; Department of Medicine, University of California, 101 The City Drive South, Orange, Irvine, CA 92868 USA; Departments of Physiology and Biomedical Engineering, and Anesthesiology, Mayo Clinic, 200 First Street SW, Rochester, MN 55905 USA; Department of Applied Physiology and Kinesiology, University of Florida, Gainesville, FL 32611 USA

## Abstract

**Introduction:**

Immobilization of hindlimb muscles in a shortened position results in an accelerated rate of inactivity-induced muscle atrophy and contractile dysfunction. Similarly, prolonged controlled mechanical ventilation (CMV) results in diaphragm inactivity and induces diaphragm muscle atrophy and contractile dysfunction. Further, the application of positive end-expiratory airway pressure (PEEP) during mechanical ventilation would result in shortened diaphragm muscle fibers throughout the respiratory cycle. Therefore, we tested the hypothesis that, compared to CMV without PEEP, the combination of PEEP and CMV would accelerate CMV-induced diaphragm muscle atrophy and contractile dysfunction. To test this hypothesis, we combined PEEP with CMV or with assist-control mechanical ventilation (AMV) and determined the effects on diaphragm muscle atrophy and contractile properties.

**Methods:**

The PEEP level (8 cmH_2_O) that did not induce lung overdistension or compromise circulation was determined. *In vivo* segmental length changes of diaphragm muscle fiber were then measured using sonomicrometry. Sedated rabbits were randomized into seven groups: surgical controls and those receiving CMV, AMV or continuous positive airway pressure (CPAP) with or without PEEP for 2 days. We measured *in vitro* diaphragmatic force, diaphragm muscle morphometry, myosin heavy-chain (MyHC) protein isoforms, caspase 3, insulin-like growth factor 1 (IGF-1), muscle atrophy F-box (MAFbx) and muscle ring finger protein 1 (MuRF1) mRNA.

**Results:**

PEEP shortened end-expiratory diaphragm muscle length by 15%, 14% and 12% with CMV, AMV and CPAP, respectively. Combined PEEP and CMV reduced tidal excursion of segmental diaphragm muscle length; consequently, tidal volume (VT) decreased. VT was maintained with combined PEEP and AMV. CMV alone decreased maximum tetanic force (Po) production by 35% versus control (*P* < 0.01). Combined PEEP and CMV did not decrease Po further. Po was preserved with AMV, with or without PEEP. Diaphragm muscle atrophy did not occur in any fiber types. Diaphragm MyHC shifted to the fast isoform in the combined PEEP and CMV group. In both the CMV and combined PEEP and CMV groups compared to controls, IGF-1 mRNAs were suppressed, whereas Caspase-3, MAFbx and MuRF1 mRNA expression were elevated.

**Conclusions:**

Two days of diaphragm muscle fiber shortening with PEEP did not exacerbate CMV-induced diaphragm muscle dysfunction.

## Introduction

Studies in both animals [1,2] and humans [3,4] have demonstrated that complete diaphragm muscle inactivity due to controlled mechanical ventilation (CMV) induces a rapid decline in diaphragm muscle function associated with muscle fiber injury [1] and fiber atrophy [3,5]. This phenomenon is commonly termed ventilator-induced diaphragmatic dysfunction (VIDD) [6]. Conversely, partial diaphragm muscle activation with assist-control mechanical ventilation (AMV) [7] or adaptive servo-ventilation (ASV) [8] preserves diaphragmatic function and mitigates CMV-induced diaphragm muscle injury [9] and atrophy [8].

In addition to CMV-induced diaphragm muscle inactivity, it is unknown whether shortening of diaphragm muscle fiber from the application of positive-end expiratory airway pressure (PEEP) during mechanical ventilation influences diaphragm muscle structural and contractile properties. This is important because, in rat hindlimb skeletal muscle immobilized with the muscle in a shortened position, protein synthesis has been observed to decrease more rapidly than when the muscle was stretched beyond its resting length [10]. Further, in other studies of hindlimb immobilization with plaster cast [11] or suspension [12], researchers have reported that greater muscle atrophy occurs when muscles are immobilized at a fiber length less than the resting length.

In the diaphragm, PEEP application increases lung volume above its equilibrium volume and shortens diaphragm muscle fiber [13]. PEEP is an important component in the management of patients receiving mechanical ventilation. In patients with acute respiratory distress syndrome (ARDS), PEEP improves oxygenation and mitigates lung injury by preventing the closing and opening of the collapsed alveoli [14,15]. In patients with chronic obstructive pulmonary disease, PEEP decreases the work of breathing by counteracting the threshold load [16]. However, the benefits of PEEP might be offset by its possible deleterious effect of shortened diaphragm muscle fiber. When PEEP is applied with CMV, the diaphragm constantly cycles passively from its shortened muscle fiber length. With AMV, the application of PEEP leads to diaphragm muscle contractions, also from a shortened muscle fiber length above its resting lung volume.

The mechanisms responsible for CMV-induced diaphragmatic dysfunction are complex. Nonetheless, CMV-induced oxidative stress increases in the diaphragm [17-20], leading to myonuclear apoptosis [21,22] and protein degradation via numerous proteolytic systems, including autophagy [23], calpains [4,5,24], caspases [24] and the ubiquitin proteasome system [4,25,26]. CMV-induced molecular changes in the diaphragm include the overexpression of genes associated with protein degradation—muscle atrophy F-box (MAFbx or atrogin 1) and muscle ring finger protein 1 (MuRF1)—are accompanied by simultaneous suppression of genes associated with protein synthesis (insulin-like growth factor 1 (IGF-1)) [25,27].

It is established that immobilization of hindlimb skeletal muscles in a shortened position accelerates fiber atrophy; however, it is unclear whether the application of PEEP will exaggerate the diaphragm muscle changes induced by CMV or AMV alone. Therefore, in this investigation, we tested the hypothesis that, compared to CMV or AMV without PEEP, the combination of PEEP and CMV or AMV would accelerate ventilator-induced activation of proteolytic activity, resulting in a more rapid development of diaphragm muscle atrophy and contractile dysfunction.

## Material and methods

### Subjects, animal preparation, surgical procedures and experimental protocol

The study received approval of the Research and Development Subcommittee on Animal Studies of the Veterans Affairs Long Beach Healthcare System. A total of 50 pathogen-free male New Zealand White rabbits were studied. Four animals were employed for PEEP titration, four animals for determination of segmental length changes of diaphragm muscle fiber with PEEP application and forty-two animals for 2 days of mechanical ventilation with or without PEEP.

Surgeries were performed while the animals were under general anesthesia with an intramuscular mixture of ketamine hydrochloride 35 mg/kg and xylazine 5 mg/kg. Maintenance of anesthesia (intramuscular ketamine hydrochloride 2 mg/kg and xylazine 0.2 mg/kg) was provided every 20 minutes as needed. Monitoring of the depth of anesthesia was based on the absence of jaw tone. The trachea was cannulated with a tracheostomy tube (4 mm inner diameter, 6 cm long). The external jugular vein was cannulated for continuous infusion of fluids and intravenous medications. The common carotid artery was cannulated for blood pressure monitoring (Grass model P23XL; Astro-Med, West Warwick, RI, USA) and for heart rate monitoring and arterial blood sampling, as previously reported [1,7]. For animals receiving 2 days of mechanical ventilation, a feeding tube was inserted into the stomach via a small incision in the esophagus.

**Figure 1:**
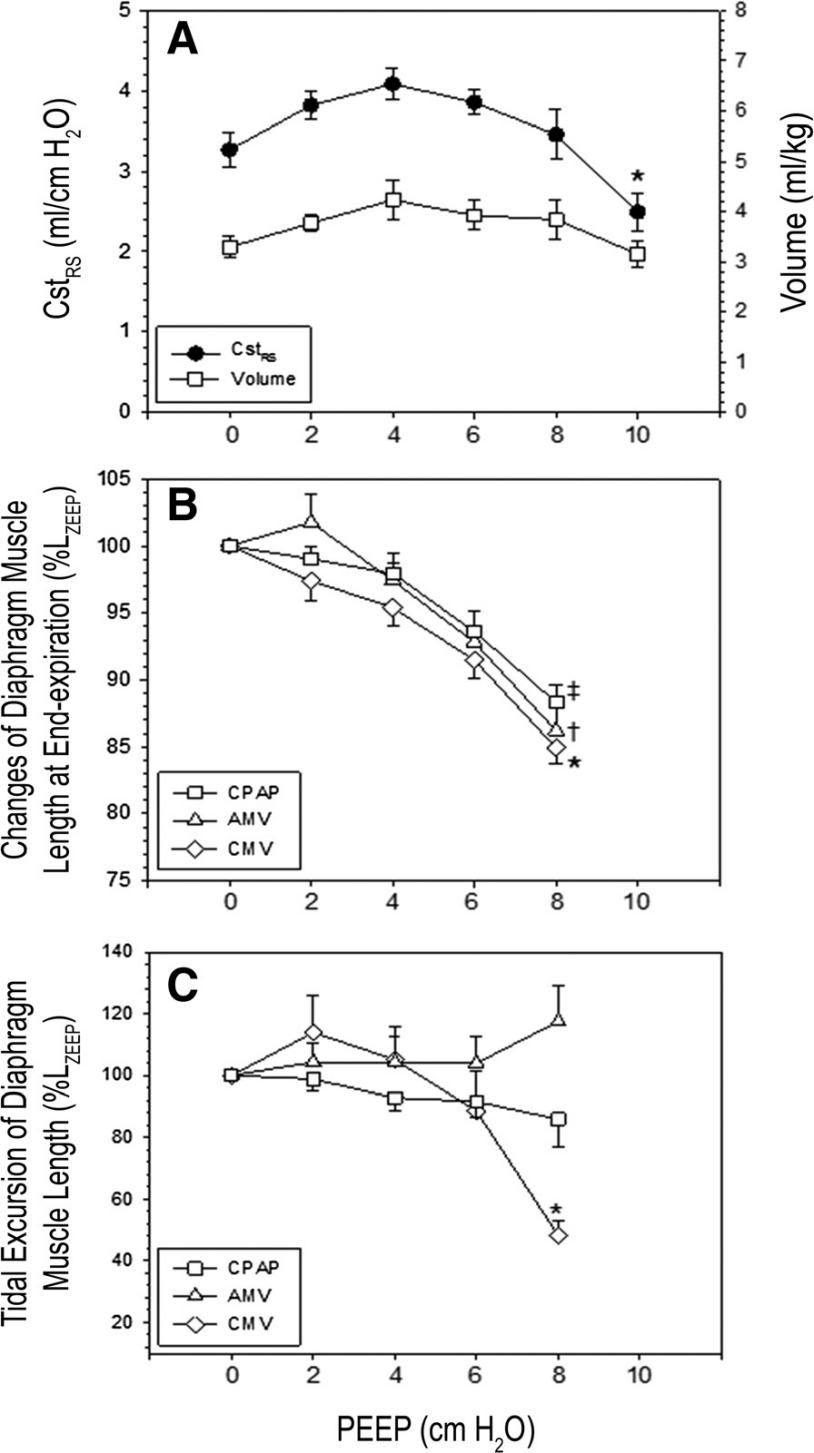
**Data gathered at varying positive end-expiratory pressure levels.** **(A)** Static compliance of the respiratory system (Cst_RS_) and volume normalized for body weight. **(B)** Changes of costal segmental diaphragm muscle length at end expiration expressed as a percentage of that at positive end-expiratory airway pressure (PEEP) of 0 cmH_2_O (L_ZEEP_). **(C)** Tidal excursion of segmental diaphragm muscle length expressed as a percentage of that at L_ZEEP_. AMV, Assist-control mechanical ventilation; CMV, Controlled mechanical ventilation; CPAP, Continuous positive airway pressure. Paralysis was incorporated only in experimental results shown in (A). Values are mean ± SE (*n* = 4 animals in each experiment). (A) *P < 0.05 PEEP of 10 cmH_2_O compared with 8, 6, 4 and 2 cmH_2_O. (B) ‡*P* < 0.01 CPAP of 8 cmH_2_O compared with CPAP of 6, 4, 2 and 0 cmH_2_O; †*P* < 0.01 AMV with PEEP of 8 cmH_2_O compared with PEEP of 6, 4, 2 and 0 cmH_2_O; *P < 0.01 CMV with PEEP of 8 cmH_2_O compared with PEEP of 6, 4, 2 and 0 cmH_2_O. (C) *P < 0.01 CMV with PEEP of 8 cmH_2_O compared with AMV with PEEP of 8 cmH_2_O and with various PEEP and CPAP levels (0 to 6 cmH_2_O).

For PEEP titration, animals were paralyzed with cisatracurium (0.1 mg/kg intravenously followed by infusion of 3 μg/kg/min). PEEP was titrated to its physiological limits, defined as pressure applied without the induction of lung overdistension or circulatory compromise in a paralyzed animal. For the PEEP titration protocol, pressure-cycled CMV (Puritan Bennett model 840 ventilator; Covidien, Carlsbad, CA, USA) was applied with set inspiratory pressure (IP) to deliver a very small tidal volume (VT) of about 4 ml/kg (animal resting breathing VT; see Table 1). VT was obtained by integrating the flow signal, measured using a heated pneumotachograph (model 8300B; Hans Rudolph, Shawnee, KS, USA) connected to the tracheostomy tube. The inspiratory time (TI) was set to approximately that of an average TI of spontaneous breaths, and the ventilator rate was set at 40 breaths/min. PEEP was applied at increasing increments of 2 cmH_2_O for 5 minutes each until static compliance of the respiratory system (Cst_RS_) decreased below that of control (Figure 1A). Cst_RS_ was calculated as the ratio of changes in volume to changes in airway plateau pressure (P_plat_) minus applied PEEP. P_plat_ was obtained using the end-inspiratory occlusion technique held for 1 second by pressing the inspiratory hold button of the ventilator. An average of three P_plat_ values obtained at 1-minute intervals and average VTs measured prior to airway occlusion were used for Cst_RS_ calculation. As shown in Figure 1A, Cst_RS_ decreased significantly at PEEP of 10 cmH_2_O; therefore, for the subsequent experiments carried out to assess the effects of PEEP on diaphragm muscle function (see below), we applied a PEEP of 8 cmH_2_O combined with CMV or AMV for 2 days, taking into account the set VT above PEEP of 6 to 8 ml/kg body weight, similar to that used in clinical practice but higher than that used for PEEP titration. Mean arterial pressure (MAP) was maintained at 70 mmHg, if necessary by boluses of intravenous fluid infusion.

**Table 1:** **Peak inspiratory airway pressure, applied positive end-expiratory airway pressure, breathing pattern and timing on various modes of mechanical ventilation** ^**a**^

**Control**	**CPAP**	**CPAP-8**	**AMV**	**AMV-8**	**CMV**	**CMV-8**
Peak inspiratory airway pressure (cmH_2_O) above PEEP						
−0.8 ± 0.2	3.2 ± 0.3	3.7 ± 0.6	8.2 ± 0.3^b^	7.7 ± 0.3^b^	8.1 ± 0.2^b^	7.4 ± 0.3^b^
Applied positive end-expiratory airway pressure (cmH_2_O)						
N/A	N/A	8.3 ± 0.2	N/A	8.1 ± 0.1	N/A	8.4 ± 0.3
Tidal volume (ml/kg)						
3.6 ± 0.1^b^	5.8 ± 0.6	6.4 ± 0.6	7.5 ± 0.3	7.4 ± 0.5	7.5 ± 0.4	5.1 ± 0.2^c^
Respiratory frequency (breaths/min)						
51.6 ± 4.8^b^	36.6 ± 2.2	29.8 2.8	30.4 ± 2.9	28.7 ± 2.9	40.1 ± 0.1	40.0 ± 0.1
Minute ventilation (L/min)						
0.6 ± 0.1	0.8 ± 0.1	0.7 ± 0.1	0.8 ± 0.1	0.7 ± 0.1	1.2 ± 0.1^c^	0.7 ± 0.0
Inspiratory time (seconds)						
0.40 ± 0.04	0.58 ± 0.02^c^	0.51 ± 0.03	0.47 ± 0.01	0.48 ± 0.01	0.48 ± 0.01	0.47 ± 0.01
Duty cycle						
0.35 ± 0.07	0.35 ± 0.02	0.25 ± 0.03	0.24 ± 0.02	0.23 ± 0.03	0.32 ± 0.01	0.32 ± 0.01

^a^Values are mean ± SE average of those after 1 and 2 days of various modes of mechanical ventilation (*n* = 6 animals in each group). AMV, Assist-control mechanical ventilation; CMV, Controlled mechanical ventilation (numerical value of 8 next to the ventilatory mode denotes the set CPAP or PEEP of 8 cmH_2_O); CPAP, Continuous positive airway pressure; N/A, Not applicable; PEEP, Positive end-expiratory airway pressure; VT, Tidal volume. Peak inspiratory airway pressure: ^b^ *P* < 0.01 compared with CPAP and CPAP-8; VT: ^b^ *P* < 0.01 compared with CPAP, CPAP-8, AMV, AMV-8 and CMV. ^c^ *P* < 0.05 compared with CMV, AMV-8 and AMV. Respiratory frequency: ^b^ *P* < 0.01 compared with CPAP, CPAP-8, AMV and AMV-8. Total minute ventilation: ^c^ *P* < 0.05 compared with control, CPAP-8, AMV-8 and CMV-8. Ti: ^c^ *P* < 0.05 compared with control, AMV, AMV-8, CMV and CMV-8.

### Determination of *in vivo* segmental length changes of diaphragm muscle fiber

*In vivo* end-expiratory and tidal excursion of segmental length changes of diaphragm muscle fibers at various levels of applied PEEP were estimated using sonomicrometry (model 120; Triton Technology, San Diego, CA, USA). While the animals were under general anesthesia, a pair of wire electrodes were implanted into the left costal hemidiaphragm using a midline laparotomy to monitor diaphragm muscle electrical activity (Grass; Astro-Med) to ensure its complete suppression during CMV. A pair of piezoelectric crystals (1 mm in diameter; Triton Technology) were positioned about 1 cm apart in the midcostal region of the right hemidiaphragm. Carefully aligned along the longitudinal axis of muscle fibers, the crystals were held in place with purse-string sutures. After implantation of the length transducers, the abdomen was tightly sutured in two layers [28]. The distance between the transducer pair was sampled at a rate of 1,537 Hz using a sonomicrometer. The output from the sonomicrometer was a DC signal proportional to length [29].

For three modes of ventilation (spontaneous breathing (CPAP), AMV or CMV), PEEP levels of 0, 2, 4, 6 and 8 cmH_2_O were applied in random order for 5 minutes each. Ventilator settings were as previously described [1,7]. Briefly, a pressure-cycled ventilator (840 Puritan Bennett; Covidien) equipped with a neonatal ventilator circuit and humidified inspired gas was employed. The inspired oxygen fraction was set to maintain arterial oxygen tension of 60 mmHg or greater. With CMV and AMV, with and without PEEP, the IP was set to produce a volume of approximately 6 to 8 ml/kg. TI was set approximate to that of spontaneous breathing. For CMV, the respiratory rate was adjusted to completely suppress spontaneous diaphragm muscle contractions based on monitoring of electrical activity. With AMV, the respiratory rate was set at 4 breaths/min. Flow trigger sensitivity was set at 1 L/min. For a given PEEP, end-expiratory and tidal excursion of segmental diaphragm muscle fiber length were expressed as the percentage of that measured at PEEP of 0 cmH_2_O (L_ZEEP_) (Figures 1B and 1C).

### Mechanical ventilation: positive end-expiratory pressure

Animals were randomized into seven groups with six animals in each group: CMV and AMV with and without PEEP of 8 cmH_2_O to contrast between complete and partial diaphragm inactivation, respectively; CPAP of 0 and 8 cmH_2_O as controls, respectively; and a sham surgical control group. The ventilator settings for CMV and AMV were identical to those employed for determination of segmental length changes of diaphragm muscle fiber. The surgical control mice were killed on the day of surgical procedures, and the other mice were killed after 2 days of ventilation.

### Animal monitoring during mechanical ventilation

In those animals that underwent 2 days of mechanical ventilation, a continuous sedation with intravenous diazepam was administered [1]. In the CMV group, suppression of diaphragm muscle activity was monitored via airway pressure and flow waveform as previously described [1] and reconfirmed in sonomicrometry experiments in which diaphragm electrical activity was monitored. Ringer lactate (80 ml/kg/day) was infused intravenously; however, with the application of 8 cmH_2_O PEEP, administration of additional fluids was necessary to maintain a MAP of 70 mmHg or greater. The lungs of animals without PEEP application were inflated with a set of IP to produce a volume of 12 ml/kg for five consecutive breaths every 15 minutes to prevent atelectasis. Liquid nutrition (100 kcal/kg/day, F3978SP; BioServ, Frenchtown, NJ, USA) was administered via a feeding tube. Blood pressure, heart rate and rectal temperature, as well as airway pressure, flow and volume, were monitored continuously as previously reported [1,7]. End-tidal partial pressure of carbon dioxide (PaCO_2_) was monitored at the proximal end of the tracheostomy tube (Capstar-100; CWE, Ardmore, PA, USA). At the end of 24 hours and 48 hours, breathing pattern and timing were measured and averaged over 30 seconds. Respiratory timing was obtained from the flow signal. Respiratory frequency was calculated as 60 divided by total breath cycle (Ttot) duration, total minute ventilation (VE) as the product of VT and respiratory frequency, and duty cycle as the ratio of TI to Ttot. Arterial blood was withdrawn for blood gas analyses every 12 hours and as needed as dictated by the end-tidal PaCO_2_. A physician or research scientist provided round-the-clock observation and animal care for the duration of the study.

### *In vitro* measurements of diaphragm muscle contractile properties

At the end of the experiments, the animals were killed with an overdose of pentobarbital sodium (100 mg/kg intravenously). The diaphragm muscle was rapidly excised from the midcostal region, with the insertion of fibers at the ribs and central tendon intact for isometric contractile measurements. Briefly, a muscle strip (approximately 5 mm wide) was obtained and mounted vertically between two platinum plate electrodes that covered the entire length of the muscle strip in a 26°C bath containing Rees-Simpson solution, through which 95% O_2_/5% CO_2_ was continuously aerated, maintaining a pH of 7.40 [1]. The rib end of the muscle was clamped, and its central tendon was attached to the lever arm of a Cambridge system (model 300B; Aurora Scientific, Aurora, ON, Canada) to allow adjustment of muscle length. The muscle was stimulated using 1.5-ms monophasic rectangular pulses delivered via a current amplifier (Mayo Foundation, Engineering Section, Rochester, MN, USA) controlled by a Grass stimulator (model S88; Astro-Med). Once the maximal stimulus intensity and optimal muscle length (Lo) for force production were determined (at 50 Hz and train of 500 milliseconds), peak twitch tension (Ptw), time-to-peak twitch tension (TPT) and RT½ (the time for Ptw to relax to one-half Ptw) were determined at Lo from a series of contractions induced by single-pulse stimuli. Isometric forces were determined using the Cambridge system, and the signals were acquired with a data acquisition system (MetraByte DAC-16; Keithley Instruments, Cleveland, OH, USA). Force outputs were sampled at a frequency of 1,000 Hz/channel. To determine maximum isometric tetanic force (Po), the muscle strip was stimulated using 1-second trains at 10, 20, 40, 50, 75 and 100 Hz with at least a 2-minute interval between each stimulus train. Forces were normalized for muscle cross-sectional area (CSA), which was estimated using the following formula:

$$ \mathrm{Muscle}\ \mathrm{mass}\left(\mathrm{g}\right)\div \left[\mathrm{L}\mathrm{o}\ \left(\mathrm{cm}\right) \times \mathrm{Muscle}\ \mathrm{density}\left(\mathrm{g}/{\mathrm{cm}}^3\right)\right], $$

using 1.056 g/cm^3^ for muscle density.

### Determination of myofiber cross-sectional area and contractile proteins

#### Myofiber cross-sectional area

A diaphragm muscle strip was prepared and stretched to 1.5 times the resting excised muscle length and rapidly frozen in isopentane cooled to its melting point by liquid nitrogen. Ten μm serial muscle sections were cut by using a cryostat kept at -20°C (model CM1850; Leica Microsystems, Buffalo Grove, IL, USA). Immunohistochemistry was performed to identify muscle fiber types [1]. Fiber CSAs of each fiber type were measured using image-processing software (Image Pro Plus v. 4.0; Media Cybernetics, Rockville, MD, USA). Fiber proportions were then calculated for specific MyHC.

#### Contractile protein fractions

An additional segment of diaphragm muscle strip was homogenized and denatured. The homogenates were loaded into the wells of a 4% to 15% gradient Tris-HCl polyacrylamide gels (Bio-Rad Laboratories, Hercules, CA, USA) at a protein concentration of 2 μg/well. The relative proportions of myosin and actin were obtained by measuring the density of each contractile protein band and expressing it as a percentage of the total density of all proteins in that sample [7].

#### Myosin heavy-chain isoform expression

MyHC protein isoforms were separated using techniques described previously [7]. This method separates fast, MyHC_2A_, MyHC_2X_ (MyHC_2B_ was not identified in rabbits diaphragm muscle) and slow (MyHC_slow_) isoforms. At the completion of electrophoresis, gels were stained for 1 hour with R-250 Coomassie Blue and then destained using a 40% methanol/10% acetic acid solution. Gels were then digitally imaged and analyzed using ImageQuant software (Molecular Dynamics, Sunnyvale, CA, USA).

### Molecular analysis of genes associated with protein degradation

Using reverse transcription coupled with quantitative RT-PCR, we measured caspase 3, IGF-1, MAFbx, and MuRF1 mRNA together with *18S* as an alternate housekeeping gene with the Bio-Rad iQ cycler system (Bio-Rad Laboratories). The sequences, respectively, for primers of rabbit oligonucleotide (Eurofins MWG Operon) for 3′-region sense and 5′-region antisense were as follows: GCT GGA CAG TGG CAT CGA GA and AAT TCA AGG GAC GGG TCA TG for caspase 3; CCG GAG CTG TGA TCT GAG GAG and CTG AGT CTT GGG CAT GTC GGT GTG for IGF-1; CAG AAC AGC AAA ACC AAA ACT CAG and GCG ATG CCA CTCA GGG ATG T for MAFbx; and GAC TCG TGC AGA GTG ACC AA and GTG GAC TTT TCC AGC TGC TC for MuRF1.

### Statistical analysis

Group size was determined on the basis of change in diaphragmatic function (that is, Po). On the basis of our previous study [1], for minimum differences in a mean of 0.30 and expected standard deviation of residuals of 0.10, we required five animals per group to achieve a power of 0.80 and α = 0.05 (analysis of variance (ANOVA); SigmaStat version 3.01, SPSS, Chicago, IL, USA). All values are expressed as mean ± SE, unless specifically indicated. Two-way ANOVA was used to compare diaphragmatic force, MAP and arterial blood gas (ABG) among groups using the appropriate grouping variables of frequency of stimulation (diaphragmatic force) or timing (MAP, ABG) with modes of ventilation (CPAP, AMV, CMV with and without PEEP). For other variables, one-way ANOVA was used for comparisons between groups. When the *F*-value was significant, *post hoc* analysis was performed using the Tukey test for pairwise multiple comparisons. Group differences were considered significant at P ≤ 0.05.

## Results

### Diaphragmatic segmental length changes with positive end-expiratory application

The application of 8 cmH_2_O PEEP or CPAP shortened end-expiratory diaphragm muscle fiber significantly, by 15%, 14% and 12% from L_ZEEP_ with CMV, AMV and CPAP, respectively (Figure 1B). When PEEP was applied to CMV (CMV-8), it markedly reduced tidal excursion of segmental diaphragm muscle length (Figure 1C). Consequently, at a set peak inspiratory airway pressure similar to that used with CMV, VT was significantly reduced with CMV-8 (Table 1). In contrast, VT was preserved with AMV-8 (Table 1). The maintenance of tidal excursion of segmental diaphragm muscle length with AMV-8 is likely due to abdominal muscle recruitment to optimize diaphragmatic function, a form of load compensation to defend VT [30,31]. In controls, the small VT was accompanied by an elevated respiratory frequency to maintain a constant VE. With CMV-8, the preset frequency was adequate to maintain a constant VE, despite the reduced VT. In contrast, with CMV alone, the preset frequency increased VE significantly because, without PEEP, tidal excursion of segmental diaphragm muscle length was unaffected, and therefore its delivered VT (Table 1).

As shown in Table 2, to maintain the target MAP, the groups with CMV and AMV combined with PEEP received significantly larger amounts of intravenous fluids compared with the corresponding mode of ventilation without PEEP. In fact, blood pressure and gas exchange were stable throughout the 2-day study (Table 2).

**Table 2:** **Body weight, diazepam dose, total volume of fluid infused, mean arterial pressure and arterial blood gas at baseline and after 2 days with various modes of mechanical ventilation, with and without applied positive end-expiratory airway pressure** ^**a**^

	**Control**	**CPAP**	**CPAP-8**	**AMV**	**AMV-8**	**CMV**	**CMV-8**
Body weight (kg)							
Baseline	3.4 ± 0.1	3.7 ± 0.3	3.6 ± 0.4	3.7 ± 0.3	3.4 ± 0.2	3.8 ± 0.4	3.5 ± 0.2
Diazepam dose (mg/kg/h)							
		1.2 ± 0.0	1.3 ± 0.1	1.2 ± 0.1	1.3 ± 0.0	1.2 ± 0.1	1.3 ± 0.0
Total volume of fluid infused (ml/24 h)							
		408 ± 14	499 ± 30	402 ± 14	517 ± 34^b^	421 ± 20	545 ± 7^b^
Mean arterial pressure (mmHg)^c^							
Baseline	84.0 ± 1.4	88.0 ± 3.9	79.9 ± 4.0	87.2 ± 4.1	90.1 ± 5.8	86.8 ± 4.4	82.5 ± 1.2
2 days		97.9 ± 2.3	91.4 ± 4.9	95.6 ± 4.2	93.4 ± 4.3	88.4 ± 4.1	83.6 ± 3.8
Arterial blood gas (pH units)^c^							
Baseline^d^	7.45 ± 0.01	7.43 ± 0.02	7.46 ± 0.02	7.43 ± 0.02	7.45 ± 0.02	7.46 ± 0.03	7.47 ± 0.03
2 days		7.50 ± 0.04	7.48 ± 0.03	7.46 ± 0.02	7.48 ± 0.01	7.51 ± 0.03	7.49 ± 0.02
PaCO_2_ (mmHg)							
Baseline^d^	43.5 ± 2.0	44.3 ± 3.5	46.4 ± 1.5	47.6 ± 2.1	42.1 ± 2.8	47.7 ± 2.9	42.0 ± 3.2
2 days		37.2 ± 3.3	40.7 ± 2.9	35.8 ± 3.6	39.4 ± 2.2	30.5 ± 2.9	42.0 ± 1.2
HCO_3_^−^ (mEq/L)							
Baseline^d^	32.3 ± 1.8	28.5 ± 2.4	35.1 ± 1.3	32.3 ± 2.2	30.6 ± 1.8	33.6 ± 0.6	33.0 ± 2.0
2 days		28.8 ± 1.7	29.7 ± 1.3	26.0 ± 3.0	29.6 ± 1.6	24.3 ± 2.0	32.2 ± 1.7
PaO_2_/FIO_2_ (mmHg)							
Baseline^d^	379 ± 24	322 ± 10	375 ± 18	354 ± 17	367 ± 13	354 ± 21	371 ± 20
2 days		384 ± 13	389 ± 13	389 ± 15	384 ± 6	387 ± 12	383 ± 12

^a^Values are mean ± SE, except mean ± SD for body weight (*n* = 6 animals in each group). AMV, Assist-control mechanical ventilation; CMV, Controlled mechanical ventilation (numerical value of 8 next to ventilatory mode denotes the set CPAP or PEEP of 8 cmH_2_O); CPAP, Continuous positive airway pressure; HCO_3_^−^, Bicarbonate; PaCO_2_, Partial pressure of carbon dioxide in arterial blood; PaO_2_/FIO_2_, Ratio of partial pressure of oxygen in arterial blood to fraction of inspired oxygen; PEEP, Positive end-expiratory airway pressure. ^b^ *P* < 0.01 for AMV-8 and CMV-8 compared with the corresponding mode without PEEP. ^c^Data obtained at 12 hours, 24 hours and 36 hours are not shown, but they were included in statistical analysis. ^d^Baseline arterial blood gas values were obtained during spontaneous breathing while the animals were under general anesthesia.

### Isometric diaphragm muscle contractile properties

**Figure 2:**
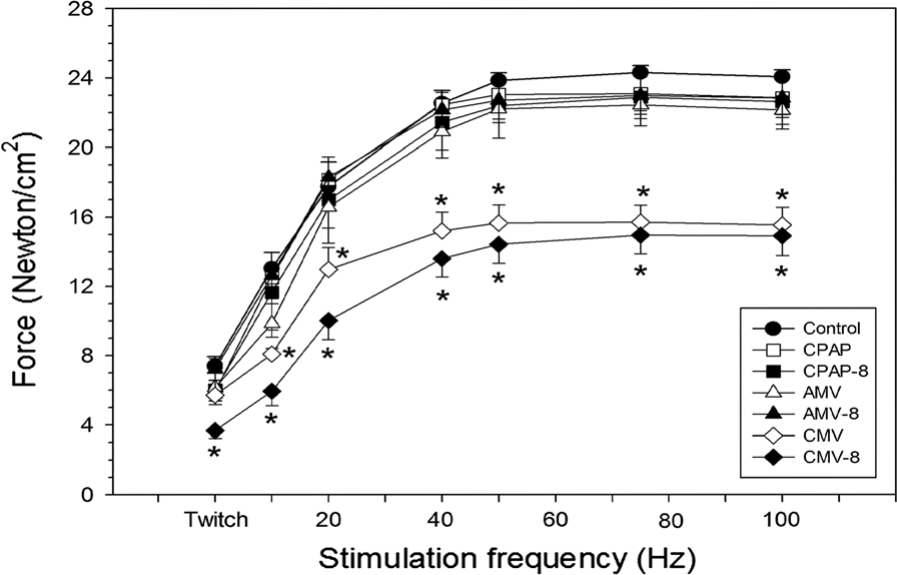
**Diaphragm muscle force–frequency relationships in control and mechanical ventilation with and without applied positive end-expiratory airway pressure.** Values are mean ± SE (n = 6 animals in each group). *P < 0.01 for controlled mechanical ventilation (CMV-8) compared with control and assist-control mechanical ventilation (AMV-8) for twitch force; CMV and CMV-8 versus corresponding modes of mechanical ventilation without and with positive end-expiratory pressure (PEEP), respectively, at stimulation frequencies of 10 to 100 Hz. The number 8 next to the mode of ventilation denotes the set CPAP or PEEP of 8 cmH_2_O. Force was normalized for muscle cross-sectional area (see Material and methods for calculation).

After 2 days of CMV or CMV-8, Po was significantly reduced compared with AMV or CPAP without or with PEEP (AMV-8, CPAP-8), respectively (Table 3). The combined CMV and PEEP did not aggravate the already-reduced Po with CMV alone. Also, PEEP did not have any effects on diaphragmatic force-generating capacity when combined with AMV or CPAP. Likewise, at all stimulation frequencies, PEEP had no additional effects on the already-reduced force with CMV alone or on the force preserved with AMV and CPAP (Figure 2).

**Table 3:** ***In vitro*** **diaphragm muscle contractile properties with various modes of mechanical ventilation, with and without applied positive end-expiratory airway pressure** ^**a**^

	**Control**	**CPAP**	**CPAP-8**	**AMV**	**AMV-8**	**CMV**	**CMV-8**
Lo (cm)	3.2 ± 0.1	3.1 ± 0.1	3.4 ± 0.1	3.2 ± 0.1	3.2 ± 0.2	2.9 ± 0.2	3.2 ± 0.1
TPT (ms)	67 ± 3	68 ± 3	73 ± 9	71 ± 3	71 ± 2	70 ± 4	66 ± 6
RT_1/2_ (ms)	84 ± 4	116 ± 11	115 ± 17	98 ± 5	96 ± 5	118 ± 8	89 ± 11
Ptw (N/cm^2^)	7.4 ± 0.5	5.9 ± 0.5	6.0 ± 0.8	6.2 ± 0.8	7.2 ± 0.6	5.7 ± 0.9	3.7 ± 0.4^b^
Po (N/cm^2^)	24.3 ± 0.4	23.3 ± 1.0	23.0 ± 1.6	22.6 ± 0.8	23.0 ± 1.1	15.8 ± 1.0^c^	15.0 ± 1.1^c^

^a^Values are mean ± SE (*n* = 6 animals in each group). Ptw and Po are normalized for cross-sectional area (see Material and methods for calculation). AMV, Assist-control mechanical ventilation; CMV, Controlled mechanical ventilation (numerical value of 8 next to ventilatory mode denotes the set CPAP or PEEP of 8 cmH_2_O); CPAP, Continuous positive airway pressure; Lo, Length at which diaphragm muscle strip produced maximal isometric tension; Po, Maximum tetanic force; Ptw, Peak twitch force; RT_1/2_, Time required for peak twitch force to relax to one-half of peak twitch force; TPT, Time from onset of muscle contraction to peak twitch force. ^b^For Ptw, *P* < 0.01 for CMV-8 compared with control and AMV-8. ^c^For Po, *P* < 0.01 for CMV compared with control, CPAP and AMV, as well as for CMV-8 compared with control, CPAP-8 and AMV-8.

### Fiber cross-sectional area, and contractile proteins

Table 4 shows that after 2 days of CMV or AMV, with or without applied PEEP, fiber CSA was unchanged compared with CPAP or CPAP-8, respectively, or with control, although a trend of reduced CSA for all fiber types was demonstrated with CMV and CMV-8. The proportion of all fiber types was not significantly different between groups (Table 4).

**Table 4:** **Diaphragm muscle fiber cross-sectional area and proportion with various modes of mechanical ventilation with and without applied positive end-expiratory airway pressure** ^**a**^

	**Control**	**CPAP**	**CPAP-8**	**AMV**	**AMV-8**	**CMV**	**CMV-8**
Fiber cross-sectional areas (μm^2^)							
MyHC_slow_	1,725 ± 80	1,581 ± 79	1,922 ± 146	1,778 ± 180	1,658 ± 150	1,404 ± 174	1,407 ± 60
MyHC_fast_	2,565 ± 214	2,478 ± 108	2,416 ± 113	2,606 ± 156	2,872 ± 193	2,016 ± 284	2,302 ± 143
Fiber proportion (%)							
MyHC_slow_	44.8 ± 3.0	41.0 ± 4.8	36.3 ± 1.8	41.5 ± 3.7	45.9 ± 3.5	37.6 ± 2.5	39.1 ± 2.0
MyHC_fast_	55.2 ± 3.0	59.0 ± 4.8	63.7 ± 1.8	58.5 ± 3.7	54.1 ± 3.5	62.4 ± 2.5	60.9 ± 2.0

^a^Values ± SE (*n* = 4 animals in each group). AMV, Assist-control mechanical ventilation; CMV, Controlled mechanical ventilation (numerical value of 8 next to ventilatory mode denotes the set CPAP or PEEP of 8 cmH_2_O); CPAP, Continuous positive airway pressure; MyHC, Myosin heavy chain.

The average total myosin pool with various modes of mechanical ventilation was similar to control, except for the CMV and AMV-8 groups, in which myosin was reduced. With the combined CMV and PEEP, MyHC isoform expressions were altered with a shift toward fast myosin (MyHC_2X_) compared with control, AMV-8 and CPAP-8 (Table 5).

**Table 5:** **Diaphragm muscle contractile proteins with various modes mechanical ventilation, with and without applied positive end-expiratory airway pressure** ^**a**^

	**Control**	**CPAP**	**CPAP-8**	**AMV**	**AMV-8**	**CMV**	**CMV-8**
Myosin and actin (% of total protein pool)							
Myosin	41.2 ± 1.0	44.5 ± 1.6	41.3 ± 1.6	41.1 ± 1.4	37.5 ± 0.9^b^	38.5 ± 1.3^b^	41.6 ± 1.5
Actin	20.2 ± 1.0	18.1 ± 1.3	19.1 ± 1.0	18.9 ± 1.6	21.7 ± 2.0	18.0 ± 1.5	17.3 ± 1.4
Myosin heavy-chain isoform expression (%)							
MyHC_slow_	35.3 ± 3.3	34.4 ± 1.3	29.9 ± 2.3	33.7 ± 1.6	30.5 ± 2.1	35.3 ± 2.0	29.5 ± 1.9
MyHC_2A_	54.4 ± 2.8	53.9 ± 1.8	60.3 ± 3.0	54.7 ± 1.4	59.0 ± 2.8	50.9 ± 2.3	55.6 ± 2.2
MyHC_2X_	10.3 ± 0.6	11.7 ± 0.6	9.8 ± 1.1	11.6 ± 0.9	10.4 ± 1.2	13.8 ± 0.4	14.9 ± 0.9^b^

^a^Values are mean ± SE (*n* = 6 animals in each group). AMV, Assist-control mechanical ventilation; CMV, Controlled mechanical ventilation (numerical value of 8 next to ventilatory mode denotes the set CPAP or PEEP of 8 cmH_2_O); CPAP, Continuous positive airway pressure; MyHC, Myosin heavy chain; PEEP, Positive end-expiratory pressure. ^b^For myosin, *P* < 0.05 compared with CPAP; ^b^for MyHC_2X_ isoform, *P* < 0.05 compared with control, CPAP-8 and AMV-8.

### Levels of transcripts associated with protein degradation and synthesis

**Figure 3:**
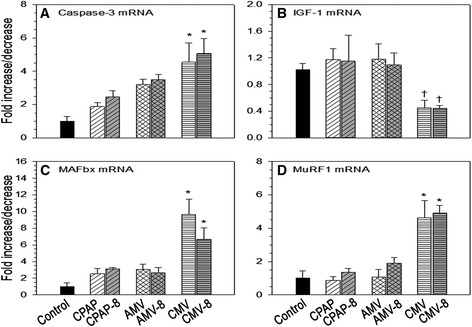
**Mechanical ventilation and applied positive end-expiratory airway pressure effects on mRNA levels. (A)** Caspase 3. **(B)** Insulin-like growth factor 1 (IGF-1). **(C)** Muscle atrophy F-box (MAFbx). **(D)** Muscle ring finger protein 1 (MuRF1). Values are mean ± SE (*n* = 6 animals per group). Caspase 3: *P < 0.01 compared with control. IGF-1: †*P* < 0.05 for CMV compared with CPAP; and for CMV-8 compared with AMV-8. MAFbx: *P < 0.01 for CMV compared with control, CPAP and AMV; and for CMV-8 compared with control. MuRF1: *P < 0.01 for CMV compared with control, CPAP and AMV; and for CMV-8 compared with control, CPAP-8 and AMV-8. AMV, Assist-control mechanical ventilation; CMV, Controlled mechanical ventilation (numerical value of 8 next to the ventilatory mode denotes the set CPAP or PEEP of 8 cmH_2_O); CPAP, Continuous positive airway pressure.

Caspase 3 mRNA tended to be elevated for all modes of mechanical ventilation, but attained statistical significance only with CMV and CMV-8 (fivefold increase compared with control). IGF-1 mRNA, transcripts of protein synthesis, were markedly reduced with CMV and CMV-8. On the contrary, molecular markers of protein degradation, MAFbx (tenfold and sevenfold increases for CMV-8 and CMV, respectively) and MuRF1 (a fivefold increase for both CMV-8 and CMV) mRNA, were markedly elevated with CMV, with and without PEEP (Figure 3).

## Discussion

PEEP is an important treatment modality in acute hypoxic respiratory failure. Most patients receiving mechanical ventilation for ARDS require PEEP of 5 to 12 cmH_2_O combined with a lung-protective ventilatory strategy to improve oxygenation and to prevent atelectrauma, which is the repetitive opening and closing of lung units [32,33]. The results of the present study confirm the rapid and profound diaphragm muscle force loss with short-term CMV, and our novel results reveal that the use of PEEP combined with CMV or AMV does not exacerbate VIDD. More specifically, our major findings are that (1) the application of 8 cmH_2_O PEEP does not exacerbate (CMV) inactivity-induced diaphragm muscle dysfunction (Table 3, Figure 2), (2) combined CMV and PEEP neither produced diaphragm muscle fiber atrophy nor enhanced the overexpression of transcripts responsible for protein degradation as previously reported with CMV alone (Figure 3) [3,27] and (3) PEEP combined with AMV does not adversely affect diaphragm muscle function following 2 days of AMV. A detailed discussion of each point follows.

### Positive end-expiratory pressure did not exacerbate diaphragm muscle dysfunction induced by controlled mechanical ventilation

Similar to previous studies [1-4], the present study confirms the deleterious effects of CMV on diaphragm contractile properties. Potential explanation for the lack of interactive effects of PEEP and CMV on diaphragmatic force production is that diaphragm muscle fiber length was not sufficiently shortened with PEEP applied or that CMV exerted maximal deleterious effects on the diaphragm, such that the use of PEEP application had no additional effects. To our knowledge, we are the first to employ a PEEP level commonly applied clinically in combination with diaphragm muscle inactivity. Therefore, only comparisons with inactive hindlimb muscles can be made. In the immobilized rat soleus and gastrocnemius muscles maintained in the shortened position for 4 weeks, muscle length decreased by 20% and 26%, respectively, from that in the neutral position [34]. Clearly, the decrease in diaphragm fiber length with the PEEP applied was less than that in the shortened rat hindlimb muscles [34]. In the present study, we observed that the combined CMV and 8 cmH_2_O PEEP decreased diaphragm muscle fiber length by 15% from that of L_ZEEP_. In a study to assess diaphragmatic shortening with lung inflation in an anesthetized, paralyzed, vagotomized dog model, Road and Leevers [13] achieved a similar degree of diaphragm muscle fiber shortening at the highest positive airway pressure applied. The application of 18 cmH_2_O positive airway pressure decreased diaphragm muscle fiber length by 13% from that of L_REEV_ [13]. Differences in the level of positive airway pressure applied to achieve a similar degree of diaphragm muscle fiber length depends on the applied load and respiratory system mechanics [13], and the latter is a function of the logarithmic value of body weight [35]. Despite species differences, the study of Road and Leevers [13] suggests that diaphragm muscle fiber shortening in our rabbit model achieved its limit. To this extent, contrasts to hindlimb muscles, potential lung overdistension and circulatory compromise constrain diaphragm muscle fiber shortening with PEEP application. A threshold of shortened diaphragm muscle fiber length may be necessary to induce alterations in contractile properties in the diaphragm, an attractive hypothesis that may be arduous to test because of the physiological limitations mentioned above.

The lack of effects of PEEP on diaphragmatic function may be due to the fact that CMV alone results in a large and significant negative impact on diaphragmatic contractile properties. This phenomenon is supported by our observation on the interactive effects of 2 days of CMV and methylprednisolone [36], as well as that of Ochala *et al*. [37] in pigs after 5 days of combined CMV with corticosteroid, neuromuscular blockade or lipopolysaccharide. In our previous study [36], Po decreased by 36% with CMV alone and by 43% with combined CMV and methylprednisolone (*P* > 0.05).

### Positive end-expiratory pressure effects on diaphragm muscle structural properties and myosin heavy-chain isoform protein expression

In the rat diaphragm, atrophy of all muscle fiber types occurred as early as 18 hours of CMV and contributed to the reduced force [5]. However, in pigs, diaphragm muscle fiber structure was intact after 5 days of CMV [37]. Likewise, in rabbits, we did not demonstrate diaphragm muscle fiber atrophy after 3 days of CMV [1]; hence, after 2 days of CMV, muscle fiber atrophy is less likely to occur, as shown in the present study. The CMV-induced loss of force in rabbits was attributed to diaphragm muscle fiber injury rather than to atrophy [1,36].

Hindlimb immobilization in the shortened position leads to relatively rapid adaptation of MyHC isoform expression [38]. For the reasons mentioned above, comparison was only possible with immobilized, shortened hindlimb muscles. In rat gastrocnemius muscle, at the mRNA level, MyHC_slow_ and MyHC_2A_ isoform expressions decreased, whereas MyHC_2X_ mRNA levels were unchanged. In the soleus muscle with predominant slow twitch fibers, MyHC_slow_ expression did not change; however, MyHC_2X_ increased as early as 2 days of immobilization in the shortened position [39]. Our findings in the combined CMV and PEEP, MyHC isoform transformation mimicked that for the shortened, immobilized rat soleus muscle (Table 5). Whereas fiber-type composition of diaphragm muscle differs from that of soleus, 90% of the rabbit diaphragm muscle fibers (MyHCslow plus MyHC2A isoform) are composed predominantly of fatigue-resistant fibers, and PEEP led to a measurable transition toward the fast MyHC isoform.

### Positive end-expiratory pressure effects on molecular markers of protein synthesis and degradation

Oxidative stress in skeletal muscle fibers has been shown to depress protein synthesis and to promote proteolysis [40]. Oxidation of diaphragm protein at a molecular weight of 200 kDa (that is, myosin heavy chain oxidation) increased with CMV, with and without PEEP (data not shown), with concomitant suppression of IGF-1 mRNA [27], the gene responsible for protein synthesis via the Akt/mTOR signaling pathway [41]. Activation of proteolysis was evidenced by the overexpression of caspase 3 mRNA [42] (Figure 3). Caspase 3 plays an important role in CMV-induced protein degradation. The disrupted myofibrils resulting from the activation of proteases (lysosomal, caspase and calpain) are susceptible to enhanced proteolysis by the ubiquitin-proteasome system [43]. PEEP had no additional effects on protein oxidation, suppression of protein synthesis or upregulation of proteolysis.

### Positive end-expiratory pressure effects in partially contracting diaphragm

After 2 days of AMV, diaphragm muscle force was preserved. As with CMV, PEEP had no deleterious effect on preserved force. The results of our present study are consistent with our previously reported data, in which diaphragmatic force was preserved after 3 days of AMV in comparison to CMV [7]. Diaphragm muscle electrical activity was maintained between 30% and 80% of control [44]. Similarly, in pigs subjected to adaptive servo-ventilation (ASV) for 3 days, diaphragmatic force was preserved [8]. Conversely, Hudson *et al*. [45] showed that, in rats, 18 hours of high-level pressure supported decreased diaphragmatic force, but at a slower rate than with CMV. High-level pressure support also activated calpain, caspase 3 and 20S proteasome [45]. In the present study, caspase 3 mRNA tended to increase after 2 days of AMV with and without PEEP. Differences between our study and that of Hudson *et al*. [45] may be due to differences in the degree of diaphragm muscle activation or in the animal species utilized.

### Critique of the experimental methods

Diaphragm muscle fiber shortening was not measured continuously for 2 days. Prolonged placement of the sonomicrometer transducers was not technically feasible, because manipulation of the animals for animal care enhanced the noise-to-signal ratio and accurate measurement of segmental length changes was impossible. However, PEEP of 8 cmH_2_O shortened diaphragm muscle fibers to their physiological limits as Cst_RS_ decreased, and, in all of the animals, application of PEEP to CMV and AMV required significantly larger amounts of fluids to maintain the target MAP. PEEP was applied for a short time (2 days only); the effects of long-term PEEP for more than 2 days, even at the modest level employed in this study, are unknown.

This study was performed in healthy animals only. By using a healthy animal model, confounding factors (for example, cytokines) [46] on the effect of PEEP on diaphragmatic function can be isolated. In the present study, we employed one level of 8 cmH_2_O PEEP. This level of PEEP shortened diaphragm muscle fiber comparable to 18 cmH_2_O of PEEP in dogs [13]; yet, when combined with either CMV or AMV, PEEP had no detectable harmful effects on diaphragmatic function. Studying low PEEP levels is unlikely to show any influence on diaphragm contractile properties, whereas very high PEEP levels may induce volutrauma.

## Conclusions

In this observational study, short-term application of 8 cmH_2_O PEEP to CMV or AMV did not exacerbate diaphragmatic dysfunction or induce diaphragmatic force loss, respectively. Similarly, PEEP by itself did not produce alterations in structural or molecular properties. The lack of PEEP effects on the diaphragm is likely related to insufficient diaphragm muscle fiber shortening due to the associated physiological limitations of alveolar overdistension and circulatory compromise at higher PEEP levels or that CMV induced profound effects on the diaphragm such that additional insult of PEEP was undetectable.

The rapid and profound development of diaphragm muscle weakness remains problematic following diaphragm muscle disuse with CMV. In view of the inconsistent results between studies regarding partial diaphragm muscle activation, the degree of diaphragm muscle activity sufficient to preserve diaphragm muscle function or its long-term effects remains to be investigated.

In clinical practice, PEEP of 8 to 10 cmH_2_O is considered a modest level of PEEP. A high levels of PEEP (mean 15 cmH_2_O, range 18 to 24 cmH_2_O) has been applied in the management of patients with ARDS [47], but, to our knowledge, the degree of diaphragm muscle fiber shortening or the effects on diaphragm muscle function has not been evaluated. In the present study, we demonstrate that the application of 8 cmH_2_O PEEP to conventional modes of mechanical ventilation appears safe and has no deleterious influence on diaphragm muscle force-generating capacity.

## Key messages

- At the level of physiological limits, PEEP did not aggravate CMV-induced diaphragmatic dysfunction or proteolysis.
- At the level of physiological limits, PEEP had no effect on diaphragm function in partially contracting diaphragm with AMV.

## Abbreviations

ABG: Arterial blood gas
AMV: Assist-control mechanical ventilation
ASV: Adaptive servo-ventilation
CMV: Controlled mechanical ventilation
CPAP: Continuous positive airway pressure
Cst_RS_: Static compliance of the respiratory system
IGF-1: Insulin-like growth factor 1
IP: Inspiratory pressure
Lo: Optimal muscle length
L_ZEEP_: Segmental diaphragm muscle fiber length at 0 cmH_2_O positive end-expiratory airway pressure
MAFbx: Muscle atrophy F-box (atrogin)
MAP: Mean arterial pressure
MuRF1: Muscle ring finger protein 1
MyHC: Myosin heavy chain
PEEP: Positive end-expiratory airway pressure
Po: Maximum isometric tetanic force normalized for muscle cross-sectional area
P_plat_: Airway plateau pressure
Ptw: Twitch force normalized for muscle cross-sectional area
RT½: Time for twitch force to relax to one-half twitch force
TI: Inspiratory time
TI/Ttot: Duty cycle
TPT: Time to peak twitch tension
Ttot: Total breath cycle
VE: Total minute ventilation
VIDD: Ventilator-induced diaphragmatic dysfunction
VT: Tidal volume
